# Enhanced Amikacin Diffusion With Ultrasound and Microbubbles in a Mechanically Ventilated Condensed Lung Rabbit Model

**DOI:** 10.3389/fphar.2019.01562

**Published:** 2020-01-16

**Authors:** Fabien Espitalier, François Darrouzain, Jean-Michel Escoffre, David Ternant, Eric Piver, Ayache Bouakaz, Francis Remerand

**Affiliations:** ^1^UMR 1253, iBrain, Université de Tours, Inserm, Tours, France; ^2^Pôle Anesthésie Réanimations, Hôpital Trousseau, CHRU de Tours, Tours, France; ^3^Laboratoire de Pharmacologie-Toxicologie, Hôpital Bretonneau, CHRU de Tours, Tours, France; ^4^Équipe PATCH, EA 7501 GICC, Tours, France; ^5^Laboratoire de Biochimie, Hôpital Trousseau, CHRU de Tours, Tours, France

**Keywords:** sonoporation, ultrasound, microbubbles, antibiotic therapy, lung, amikacin

## Abstract

The poor diffusion of intravenous antibiotics in lung tissue makes nosocomial pneumonia challenging to treat, notably in critical patients under mechanical ventilation. The combination of ultrasound and microbubbles (USMB) is an emerging method for non-invasive and targeted enhancement of uptake of various drugs in several organs. This study aims to evaluate if USMB may increase amikacin concentration in condensed lung tissues in a mechanically ventilated rabbit model. When applied 60 or 160 min after the beginning of an intravenous amikacin infusion, USMB increased amikacin concentration in the condensed lung tissue by 1.33 (*p* = 0.025) or 1.56-fold (*p* = 0.028) respectively. When applied 70 min after the beginning of an intravenous amikacin infusion, USMB increased amikacin concentration in the muscle tissue by 2.52 (*p* = 0.025). In conclusion, this study demonstrates that USMB is a promising method for the targeted delivery of amikacin in mechanically ventilated condensed lung, thus opening new therapeutic fields against lung infections.

## Introduction

Hospital-acquired and ventilator associated pneumonia (VAP) are among the most common infections in intensive care units (ICU) (Leone et al., 2018). They increase morbidity, and ICU and hospital length of stay (Rello et al., 2002; Koulenti et al., 2017). In ICU, most of pneumonia are VAP (American Thoracic Society et Infectious Diseases Society of America, 2005). They are frequently due to multidrug-resistant bacteria, which lead to poor therapeutic outcomes (Martin-Loeches et al., 2013). Aminoglycosides are recommended to treat these patients (Leone et al., 2018). Most intravenous (iv) antibiotics (*e.g.*, aminoglycosides) diffuse poorly in the lung tissue, making pneumonia challenging to treat. Moreover, the risk of systemic toxicity limits the use of high dose of iv antibiotics. To overcome these limitations, the development of innovative targeted delivery methods is required to increase the local concentration of antibiotics in infected lung tissues, while minimizing general side effects related to the systemic antibiotherapy.

Ultrasound contrast agents (UCAs) are microbubbles consisting of a gaseous core surrounded by a biocompatible shell. UCAs are currently used for diagnostic medical ultrasounds (Sennoga et al., 2017). Beyond these diagnostic practices, new promising applications of UCAs have emerged for targeted drug delivery (Kooiman et al., 2014). When UCAs and the targeted drug are intravenously co-injected and both exposed to ultrasound, it provides unprecedented possibilities for a selective therapeutic action known as sonoporation (Geers et al., 2012). Sonoporation denotes a process in which ultrasonically-activated microbubbles (USMB) pulsations induce a transient permeabilization of nearby endothelial barriers (*e.g.*, blood-tumor barrier, blood-brain barrier) (Escoffre and Bouakaz, 2018). This increases vascular permeability and so extravasation of drugs from blood into surrounding tissues (Carpentier et al., 2016; Dimcevski et al., 2016). In comparison with other physical drug delivery methods, sonoporation is a non-invasive, easy to apply, and cost-effective method. Thus, it provides a high potential for the delivery of a wide range of drugs to superficial and deep organs under the guidance ultrasonic imaging (Escoffre et al., 2013; Zhang et al., 2015; Yu et al., 2016).

Few studies investigated the therapeutic benefit of antibiotic treatments combined with USMB for improving antibiotic activity against bacterial infections (He et al., 2011; Shih et al., 2013; Lin et al., 2015; Shang et al., 2015; Dong et al., 2018; Sugiyama et al., 2018). Thus, USMB enhanced the antibacterial effect of free vancomycin against *Staphylococcus epidermidis in-vivo* biofilms compared to antibiotic treatment alone (He et al., 2011; Dong et al., 2018). In addition, USMB stimulated the release of vancomycin from vancomycin-loaded polymethyl methacrylate (PMMA) cement, thus increasing the *in-vitro* and *in-vivo* antibacterial effects on *Staphylococcus aureus* (Lin et al., 2015). Two other preclinical investigations also demonstrated that USMB improved the concentration of cefuroxime in rat prostate tissue (Shang et al., 2015) and of gentamicin in the inner ear of guinea pig (Shih et al., 2013). Recently, Sugiyama *et al*. reported that USMB significantly increased the gentamicin concentration in lung tissue in a bilateral diffuse lung infection model in spontaneously breathing mice, thus decreasing bacterial growth (Sugiyama et al., 2018). However, in clinical practice, pneumonia can show a diffuse pattern or be localized (they usually are limited to a lower lung lobe). In addition, in ICU, 80% of nosocomial pneumonia are VAP (Koulenti et al., 2017). Both localized pneumonia and lung aeration due to mechanical ventilation may limit ultrasound penetration in the lung tissue, and so USMB efficacy. Whether USMB can increase antibiotic diffusion in these conditions is a crucial preliminary question to answer, before further *in vivo* or clinical studies. Our present *in vivo* study aimed to evaluate if USMB may increase aminoglycoside diffusion in condensed lung tissue of mechanically ventilated rabbits. To establish that USMB worked well under controlled conditions, the diffusion of aminoglycoside was also evaluated in skeletal muscles.

## Materials and Methods

### Animal Preparation and Animal Model

All procedures were performed according to the ethical guidelines and were approved by the Animal Care and Regional Committee for Ethics in Animal Experiments, Val-de-Loire (2015020312576994). Forty healthy female adult New Zealand rabbits were purchased from Charles Rivers (Écully, France). Rabbits were maintained at constant room temperature with 12 h light cycle in isolation cages. At the start of the experiments, rabbits were 16–18 weeks old, weighing between 2.3 and 4.9 kg.

The rabbits were anesthetized by inhaling 3% isoflurane (Isoflurane Belamont, Paris, France) and oxygen *via* a facial oxygen mask. They were then positioned in supine position on a thermostatically controlled pad in order to maintain body temperature at about 37°C. During the whole procedure, the body temperature was monitored with a rectal temperature probe. Heart rate and pulse oximetry were continuously measured using a handheld pulse oximeter (PC-66V, Shenzhen Creative Industry, Shenzhen, China). The marginal vein of an ear was cannulated with a Jelco^®^ 22 G catheter (length 25 mm, diameter 0.95 mm; Smiths Medical International Ltd, Lancashire, UK). This catheter was used to intravenously inject atropine (100 µg/ml in saline). Subsequently, through the catheter, continuous infusions of sufentanil (1 µg/ml in saline, 3 µg/h) and propofol (5 mg/ml saline, 15 mg/h) were performed independently of rabbit body weight. *Via* a tracheotomy, the trachea was intubated (Portex^®^ tracheal tube with 2.5 mm internal diameter, without cuff; Smiths Medical International Ltd, Hythe, UK). The 40 rabbits were mechanically ventilated (Servo 300A, Siemens Elema, Solna Sweden) in a volume control support mode (inspired fraction of oxygen of 1.0, tidal volume of 8 ml/kg, no end-expiratory pressure, respiratory rate of 50 to 65 breaths per minute to maintain arterial CO_2_ in a normal range). Neuromuscular blockade was achieved using iterative iv atracurium boli (10 mg) every 45 min. The right femoral artery was cannulated using Seldinger method with a Leadercath arterial catheter (20 G, 4 cm length; Vygon, Écouen, France) for continuous arterial pressure monitoring (GE Datex-Ohmeda S/5, GE Datex-Ohmeda, Helsinki, Finland) and blood sample collection.

### Lung Condensation Model

Just before USMB, the right lung was flooded by saline. Briefly, a 19G epidural catheter (Vygon, Écouen, France) was inserted between the trachea and the intratracheal tube. The rabbit was positioned on its right side and 9 ml of saline were instilled into the trachea through the catheter. The flooding (and so the condensation) of the right lung but also the absence of flooding of the left lung were verified using lung echography (Logiq Book XP, General Electric Healthcare).

### Ultrasound Setup

Ultrasound waves for sonoporation were generated using a single-element lab-made ultrasound transducer with a center frequency of 1 MHz. The transducer had a diameter of 14 mm and was naturally focused at 25 mm. It was driven by an electrical signal generated from an arbitrary waveform generator (Agilent, Santa Clara, CA), then amplified by a power amplifier (ADECE, Artannes-sur-Indre, France). As previously described (Escoffre et al., 2010), the peak negative pressure was measured in a separate setup using a calibrated polyvinylidene fluoride needle hydrophone (diameter 0.2 mm; Precision Acoustics Ltd., Dorchester, UK) at the natural focal distance of the transducer. The lung and muscle tissues (exact anatomical locations are described in *Study Design* section) were sonicated at 1 MHz with a pulse repetition period of 100 μs, 40 cycles per pulse at peak-negative pressure of 600 kPa for a period of 3 min (Escoffre et al., 2013).

### Pharmacokinetic Analysis of Amikacin

In a set of 11 mechanically ventilated anesthetized rabbits, the beginning of amikacin infusion (Mylan SAS, Saint Priest, France, 15 mg/kg in 5 ml saline during 30 min) set as time T0. The lung was not condensed and neither microbubbles nor ultrasound were used in this pharmacokinetics (PKs) experiment. Blood samples were collected at T30 (discontinuation of amikacin infusion), T60, T90, T150, T210, and T510 minutes. Total blood samples were centrifuged (2,000 tr.min^−1^ during 10 min) after coagulation and amikacin concentrations were determined in the obtained serum by fluorescence polarization immunoassay using the chemistry analyzer Cobas Integra 400+ (Cobas Integra 400+, Roche Diagnostics, Basel Switzerland) (Domke et al, 2000). Amikacin PKs analysis allowed to set the timing of ultrasound application for the two following experiments: high serum amikacin concentration [AMK] experiment and low serum [AMK] experiment.

### Study Design

#### High [AMK] Sonoporation Experiment (N = 8 Rabbits)

Once amikacin infusion was started, the thorax, the ventral side of the abdomen, and both anterior legs of the rabbits were shaved. A right laparophrenotomy was performed. At T55 minutes the right lung was flooded.

##### Sonoporation of the Lung

At T60 minutes, the ultrasound transducer was placed in close contact with the right pulmonary lower lobe through the laparophrenotomy. Two milliliters of saline in the pleural space allowed ultrasound transmission between probe and lung tissue. An iv bolus of 400 μl of gas microbubbles (Vevo MicroMarker, FUJIFILM-VisualSonics Inc., Amsterdam, NL) was injected followed by 1 ml saline flush. One minute after microbubbles injection, ultrasound was applied to the lung using the ultrasound parameters described above. Each rabbit was then positioned in supine position and lung reaeration was checked using echography.

##### Sonoporation of the Muscle

The transducer was placed in contact with a shaved anterior leg (right or left legs were randomly chosen using a computer-generated random list) covered with ultrasound transmission gel. To ensure an accurate position, four thin needles around the transducer were used to mark the sonoporation region. At T70 minutes, an iv bolus of 400 μl of microbubbles was injected. One minute later, ultrasound was applied to the skeletal muscle of the anterior leg. Exsanguination was performed at T90 minutes.

#### Low [AMK] Sonoporation Experiment (N = 7 Rabbits)

In a separate experiment and using the USMB protocol previously described, the right condensed lung was exposed to two USMB treatments at T150 and T160 minutes. At T150 and T160, the systemic amikacin concentration was about three-fold lower than the peak of systemic amikacin concentration (**Figure 2**). Microbubbles were renewed before the second application of ultrasound. Exsanguination was performed at T170 minutes. In this experiment, the muscle was not submitted to USMB, meaning that the low [AMK] experiment was not conducted in the muscle tissue.

**Figure 1:**
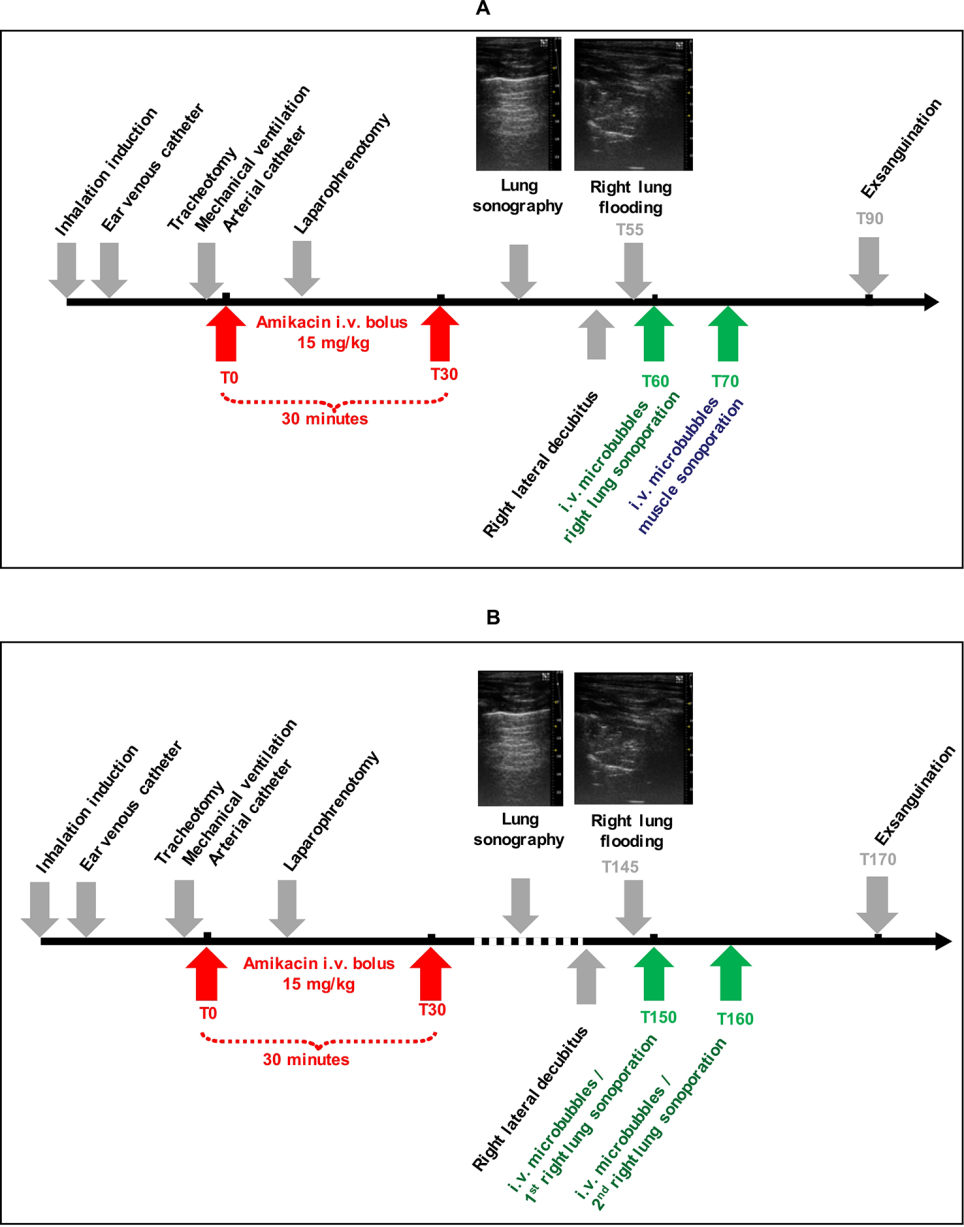
Study design with high **(A)** and low **(B)** amikacin concentrations.

The study design is presented in **Figure 1**.

### Sacrifice and Post Mortem Analysis

Each rabbit was exsanguinated *via* the arterial cannula. Through a cervicothoracic incision, both lungs were carefully dissected and separated. Their ventral and dorsal sides were photographed before removing the main vessels and bronchus. Then, two muscle samples (5 to 10 mm diameter, without serosa) were excised, one in the center of the sonicated area of the anterior leg, and the other one in the similar area on the contralateral leg (as a control sample). All lung and muscle samples were weighed with a micrometric balance. Lung and muscle samples were mechanically grinded within 5 ml of saline using Ultra-Turrax T10 device (IKA-Werke, GmbH & Co., Staufen, Germany), frozen and stored at −20°C for further analysis.

### Amikacin Concentration Assessment in Tissues

After they defrosted, grinded tissue samples were mechanically homogenized using a disperser, followed by 10 min ultrasonication. Then samples were centrifuged (2,000 tr.min^−1^ during 10 min) and amikacin was quantified in the obtained supernatant, following dilution with Roche diluent to meet the measurement accuracy range if necessary. Amikacin concentrations in tissues were determined by fluorescence polarization immunoassay (one measurement done per sample) using the chemistry analyzer Cobas Integra 400+ (Cobas Integra 400+, Roche Diagnostics, Basel Switzerland) (Domke et al, 2000). Aiming to validate the assay technique, lung and muscle supernatants containing no amikacin were used. The supernatants were divided into 10 samples. In each sample, a known dose of amikacin was added. This dose of amikacin increased from the 1st to the 10th sample. Then, in each sample, the amount of amikacin present in the supernatant was measured by the fluorescence polarization immunoassay. The same method was applied in parallel to virgin amikacin blood serum. The results obtained for each medium (blood serum, lung, or muscle supernatant) were compared with each other for each dose of amikacin added. The results of the assays in the lung supernatant and in the blood serum were commutative, which means that the assay of amikacin by the fluorescence polarization immunoassay in the lung and muscle supernatants gave exactly the same result as in the blood serum, reference medium of this technique.

### Statistics

Because of trapping of the sodium chloride used to flood the right lung, its weight was artificially increased, underestimating the amikacin concentration in this lung tissue. This “dilution effect” was corrected as follow: in a set of 14 female New Zealand rabbits without any lung flooding, right and left lungs were weighed in similar experimental conditions than previously described. The mean (standard deviation) of the ratio between right lung weight (5.803 ± 0.605 g) and left lung weight (4.047 ± 0.558 g) was 1.458 (±0.123). Amikacin concentration in the right lung was therefore corrected as follows:

$$Corrected\;{\left[AMK\right]}_{RL}=\frac{W_{RL}\times Measured\;{\left[AMK\right]}_{RL}}{W_{LL}\times 1,458}$$

with [AMK]_RL_, amikacin concentration in the right lung; W_RL_, weight of the right lung; W_LL_, weight of the left lung.

Finally, the following comparisons were made: 1) lung uptake of amikacin between sonicated and non-sonicated lung, in the presence of high [AMK] concentration, and in the presence of low [AMK] concentration, 2) muscle uptake of amikacin between sonicated and non-sonicated muscle in the presence of high [AMK] concentration. In each of these situations, the amikacin concentrations dosed in the supernatants of tissue samples were compared using a Wilcoxon test (each rabbit being its own control). A *p* value < 0.05 was considered as significant. Statistical tests were performed using SPSS 25 (IBM France statistics, Bois-Colombes, France). Neither statistical test was performed to compare amikacin lung concentration in low [AMK] concentration experiment with the one in high [AMK] concentration experiment, nor between amikacin concentration in lung and in muscle.

Because blood amikacin concentration was measured at T30, T60, T90, T150, T210, and T510, a PK modeling was needed to precisely determine the peak of blood amikacin concentration to optimize the administration of USMB in the experiments. Amikacin PKs was assessed using population PK modeling (MonolixSuite 2018, Lixoft^®^, Orsay, France). One and two-compartment models with first order absorption and elimination rate constants were tested.

## Results

### Amikacin Pharmacokinetics

**Figure 2:**
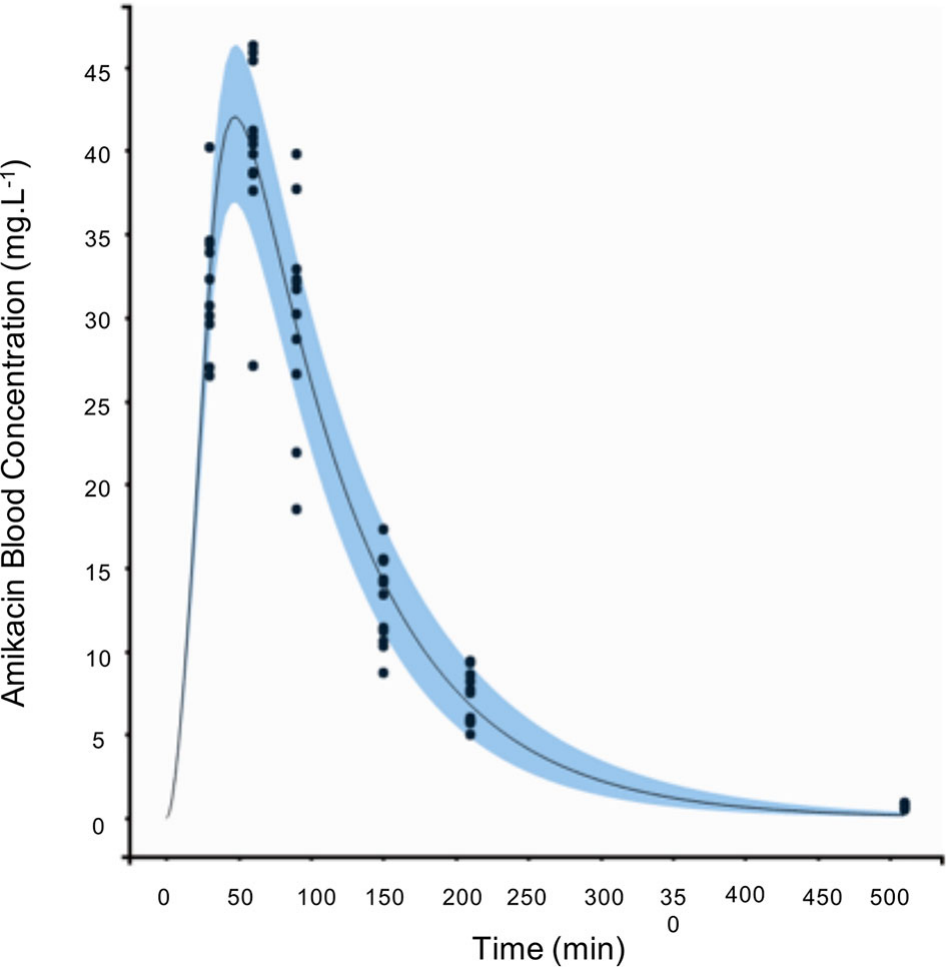
Amikacin pharmacokinetics (PKs) in mechanically ventilated anesthetized rabbits (N = 11 rabbits). T0 was set as the beginning of intravenous (iv) amikacin infusion (15 mg/kg over 30 min).

The blood amikacin concentrations were measured over time by fluorescence polarization immunoassay after iv infusion of 15 mg/kg amikacin in mechanically ventilated anesthetized rabbits. Subsequently, amikacin PKs was assessed using population PKs modeling. As shown in **Figure 2**, a one-compartment PKs model described adequately amikacin PKs. Thus, the highest amikacin concentration (40.2 ± 5.3 mg/L) was measured at 60 min after the beginning of iv amikacin infusion. Model-fitted concentration peak occurred at 48 min. Average estimates of absorption rate constant, volume of distribution, clearance, and elimination half-life constants were 0.072 min^−1^, 0.81 L, 0.01 L.min^−1^, and 56 min, respectively. Inter-subject variability of PKs parameters was <9% and none of PKs parameters was related to the body weight.

### *In-Vivo* Amikacin Delivery in Condensed Lung and Muscle Using Sonoporation

At T60 min (high [AMK] sonoporation experiment), the amikacin concentration in the right condensed lung tissue exposed to USMB was 1.33-fold higher (+33%) compared with the left non-sonicated lung (14.1 [10.3–15.4] *versus* 10.6 [8.5–14.0] μg/g; *p* = 0.025).

At T70 min, the amikacin concentration in muscle exposed to USMB was 2.52-fold higher (+152%) compared with non-sonicated muscle (6.3 [3.4–11.0] *versus* 2.5 [1.9–6.0] μg/g; *p* = 0.025).

At T160 min (low [AMK] sonoporation experiment), the amikacin concentration in the right condensed lung tissue exposed to USMB was 1.56-fold higher (+56%) compared with the left non-sonicated lung (6.1 [4.6–8.2] *versus* 3.9 [3.6–4.2] μg/g; *p* = 0.028).

**Figure 3:**
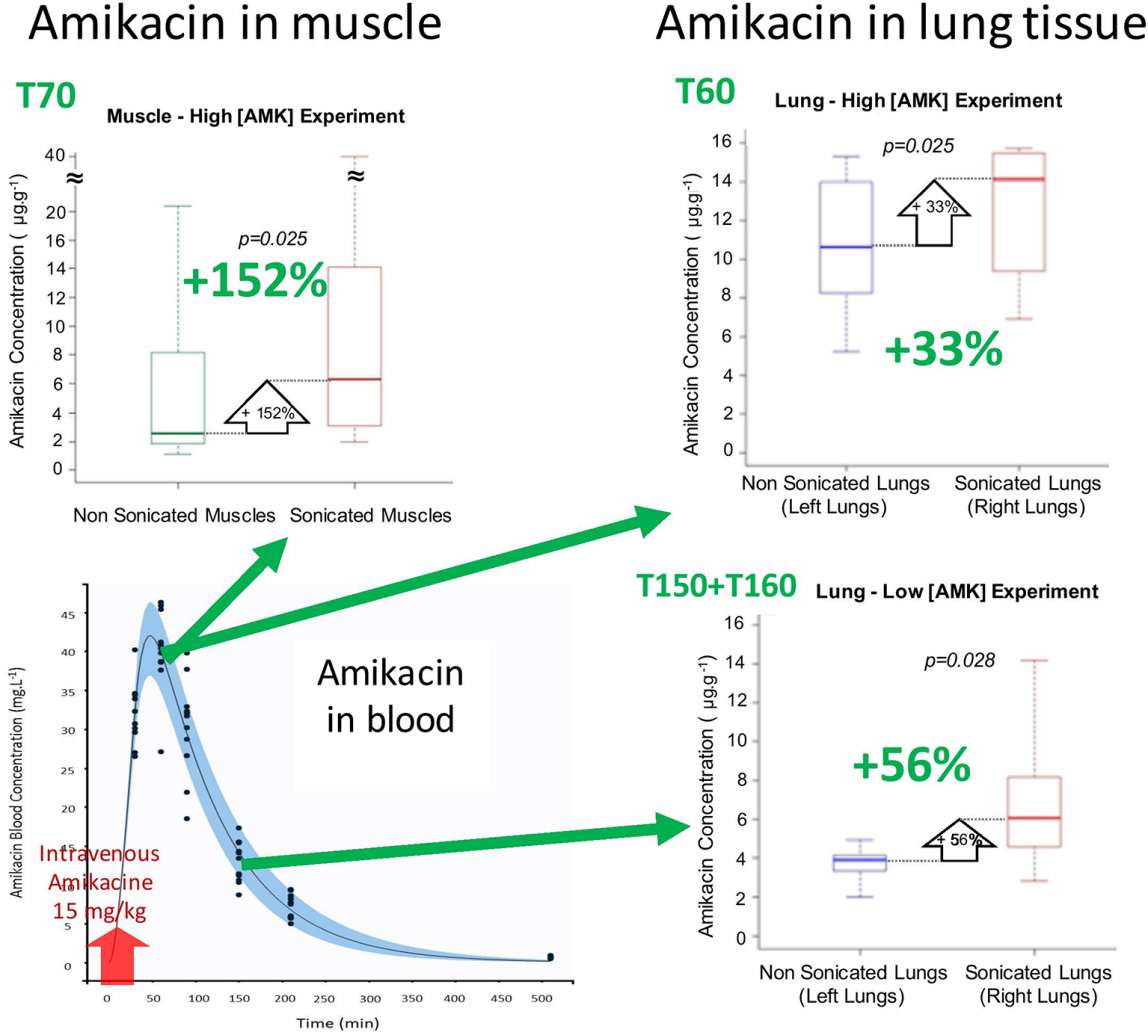
Boxplots of amikacin concentration in lung and muscle tissues (N = 7–8 rabbits/condition; each rabbit was its own control). Green arrows indicate on the PKs curve the amikacin blood concentration at the time of sonoporation.

The results are shown in **Figure 3**.

## Discussion

The present study investigated the efficacy of USMB to improve amikacin diffusion in the mechanically ventilated condensed lung in a rabbit model. It demonstrated that focal USMB significantly increased amikacin concentration in the mechanically ventilated condensed lung but also in the muscle. However, the real effect of USMB was probably underestimated in lungs because of anatomical limitations of the rabbit model and instrumental limitations of our experimental setup.

The effect of an ultrasound-based treatment applied to pneumonia may appear as unpredictable. First, the multiple air/tissue interfaces in lungs may dramatically limit ultrasound propagation into the lung and so USMB efficiency. Second, in the opposite way, in case of pneumonia, inflammatory interstitial edema increases and many alveoli are progressively filled with exudate. These conditions may ease ultrasound propagation into the lung. Third, in clinical practice, the increase in hypoxemia requires mechanical ventilation to recruit alveoli, to decrease breathing work, and to increase fraction of inspired oxygen. During mechanical ventilation, positive pressure is continuously applied in the airways to insufflate gas into the lungs and to reaerate alveoli (during spontaneous breathing, this pressure is negative during inspiration, and very low during expiration). Mechanical ventilation may therefore decrease ultrasound propagation in the lung tissue. In the present study, a model of condensed lung under mechanical ventilation was specifically developed. In our laboratory, the rabbit was the smallest available animal allowing easy mechanical ventilation. By definition, inducing a clinically relevant VAP would have required several days of mechanical ventilation, with a possible important mortality rate. This would have been time and resources consuming. So we considered it was mandatory to document the pharmacologic step before such clinical step. Two main approaches can mimic quickly a lung condensation: lung atelectasis and lung flooding. From a pathological point of view, the lung flooding model is more appropriate. Moreover, this model gives very similar echographic aspect than pneumonia in human clinical practice (see **Figure 1**). Last but not least, each rabbit was its own control, limiting interindividual variability impact. In this model, a key methodological point was the timing of both USMB and amikacin infusion. To our knowledge, the amikacin PKs has not been described in a rabbit model before. So, it had to be performed in ours. Amikacin PKs was very reproducible, since inter-individual variability was very low (< 9%), and did not depend on the rabbit weight. This PKs study allowed us to perform the USMB protocol at the highest plasmatic concentration of amikacin, in the high [AMK] experiment, when the effect should be maximal. It also allowed us to perform the USMB protocol when amikacin concentration decreased by 66%, in the so called “low [AMK] experiment.” It aimed to test if the increased diffusion was potentially dependent from the amikacin concentration.

Our main findings are USMB increased amikacin lung concentration by 33% in the high [AMK] experiment (*p = 0.025*) and by 56% in the low [AMK] experiment (*p = 0.028*), and by 152% (*p = 0.025*) in the muscle tissue. Amikacin therapeutic effect on bacteria is mainly observed during the period of high amikacin concentration. So, in the aim to optimize amikacin efficacy, the more relevant timing to apply an USMB protocol is probably the one chosen in the high [AMK] experiment, or slightly before (in theory at T48). However, our findings revealed also that USMB was effective in increasing amikacin diffusion in lung, whatever the amikacin concentration. In another approach, USMB could maintain the amikacin therapeutic effect on bacteria in the lungs while reducing its posology, in the aim to decrease its systemic toxicity.

The relative increase in amikacin concentration due to USMB was +152% in muscle tissue compared to +33 and +56% in the lung. The apparent higher efficiency of USMB in muscle than in lung tissue should be challenged: first, the spread of ultrasound is better in muscle because of the lack of tissue/gas interfaces in this tissue; second, the muscle is more superficial than lung, making it easier to sonicate; third, the accurate localization of the probe and of its beam in the muscle tissue allowed us to collect muscle tissue sample mainly in the sonicated area, leading to a better evaluation of the USMB effect on amikacin diffusion in muscle than in lung. Indeed, the real effect of USMB is probably underestimated in lungs because of anatomical limitations of the rabbit model and instrumental limitations of our experimental setup. Therefore, with appropriate ultrasonic probe for lung sonoporation, an increase of antibiotics diffusion in lung similar to the one observed in muscle should be reachable.

Due to the particular anatomy of the rabbits, with a narrow chest and a central heart, only a direct application of the probe on the right lung allowed the lung sonication while avoiding heart sonication. We presumed that if lung and heart were sonicated simultaneously, the microbubbles would have been extensively disrupted in cardiac cavities, before reaching the lung. This is the main reason why a transthoracic approach was not performed in the present study. In this rabbit model, we chose a focal application of ultrasound. The size of the probe (14 mm external diameter and a natural focus at 25 mm) allowed only limited sweeping motion once in the right hemithorax of the rabbits. Therefore, the volume of lung sonicated in our protocol with this probe (5 mm diameter beam and about 15 mm lung depth) was far less than 1 ml. It has to be compared with the median right lung weight in similar healthy female rabbits in this study (5.8 g), or with the median volume of the right lung measured on computed tomography in ten 7 month old female rabbits (30 ml) (Müllhaup et al., 2017). In the present study, the measured amikacin concentration in the whole right lung is therefore the mean of less than 1 ml of treated lung and of 5 to 30 ml of untreated lung. An accurate localization of the sonicated area could have circumvented this pitfall. This was not possible due to the cumbersome footprint of the probe, and to the respiratory lung mobility during USMB. At bedside, human lower lobes can easily be explored using transthoracic ultrasound, without visualizing the heart. So, non-invasive lung sonoporation should be feasible in clinical practice.

In mice in spontaneous ventilation, the relative increase of aminoglycoside (gentamicin) concentration in lung tissue induced by USMB was +100% (Sugiyama et al., 2018). This may appear more important than under mechanical ventilation (+33 or +56%), as showed in our study. Our results have to be challenged since they are largely underestimated (see above). Moreover, Sugiyama’s animal model and aminoglycoside studied were different from our experiments. In addition, in Sugiyama’s murine model, the whole thorax was sonicated (Sugiyama et al., 2018). Such large sonication is difficult to conceive in clinical practice. Our model was a non-infected one, so we were not able to directly demonstrate the anti-bacterial therapeutic effect related to the increase of lung amikacin concentration as recently reported with gentamicin (Sugiyama et al., 2018).

The conclusions drawn from our study should not be extended to other antibiotic classes. Indeed, the pharmacological and physicochemical properties (*e.g.*, molecular weight, spatial conformation, ionic charge, *etc*.) strongly vary from an antibiotic to another and the impact on the molecule diffusion after sonoporation cannot be predicted.

USMB is a new and original therapeutic approach. Its safety has to be explored. In theory, increasing vascular permeability may increase lung edema, and so increase hypoxemia. Whether this effect is negligible, additive, or synergistic with preexisting increased vascular permeability in inflamed lungs (induced by mechanical ventilation and/or infection) has to be questioned. At bedside, no harm has been attributed to diagnostic lung ultrasounds, while they have been largely used for more than 20 years, including in injured lungs (Lichtenstein et al., 1997). USMB seems to be safe during the hours following application: in agreement with Sugiyama’s study, pulse oximetry was not modified by lung ultrasound application in our study (data not shown). Nevertheless, delayed deleterious effects of USMB cannot be excluded. This aspect will have to be explored in further studies on this topic, with a prolonged follow-up.

## Conclusions

USMB increased amikacin concentration in mechanically ventilated condensed rabbit lung. Nevertheless, further technological developments are still required to potentiate this effect, and to validate the efficacy and the safety of therapeutic protocol in a rabbit lung infection model. Future works should study if USMB could improve the efficacy of current antibiotherapy for the treatment of pulmonary diseases, specifically in hospital-acquired and ventilator associated pneumonia.

## Data Availability Statement

The datasets generated for this study are available on request to the corresponding author.

## Ethics Statement

The animal study was reviewed and approved by Animal Care and Regional Committee for Ethics in Animal Experiments, Val-de-Loire, France (2015020312576994).

## Author Contributions

AB, FR, J-ME, and FE conceived the project and the design of experiments. FE and FR performed the experiments. FE, EP, and FD performed the amikacin concentration dosing. FD and DT performed the modeling of the pharmacokinetics of amikacin. FE and FR analyzed the results, and wrote the manuscript. All authors reviewed the manuscript.

## Funding

This work was supported by institutional funds from Inserm and Université de Tours.

## Conflict of Interest

The authors declare that the research was conducted in the absence of any commercial or financial relationships that could be construed as a potential conflict of interest.

The handling editor is currently organizing a Research Topic with one of the authors J-ME, and confirms the absence of any other collaboration.
